# Effect of Tai Chi Training on Dual-Tasking Performance That Involves Stepping Down among Stroke Survivors: A Pilot Study

**DOI:** 10.1155/2017/9134173

**Published:** 2017-11-15

**Authors:** Wing-Nga Chan, William Wai-Nam Tsang

**Affiliations:** Department of Rehabilitation Sciences, The Hong Kong Polytechnic University, Kowloon, Hong Kong

## Abstract

Descending stairs demands attention and neuromuscular control, especially with dual-tasking. Studies have demonstrated that stroke often degrades a survivor's ability to descend stairs. Tai Chi has been shown to improve dual-tasking performance of healthy older adults, but no such study has been conducted in stroke survivors. This study investigated the effect of Tai Chi training on dual-tasking performance that involved stepping down and compared it with that of conventional exercise among stroke survivors. Subjects were randomized into Tai Chi (*n* = 9), conventional exercise (*n* = 8), and control (*n* = 9) groups. Those in the former two groups received 12-week training. Assessments included auditory Stroop test, stepping down test, and dual-tasking test involving both simultaneously. They were evaluated before training (time-1), after training (time-2), and one month after training (time-3). Tai Chi group showed significant improvement in the auditory Stroop test from time-1 to time-3 and the performance was significantly better than that of the conventional exercise group in time-3. No significant effect was found in the stepping down task or dual-tasking in the control group. These results suggest a beneficial effect of Tai Chi training on cognition among stroke survivors without compromising physical task performance in dual-tasking. The effect was better than the conventional exercise group. Nevertheless, further research with a larger sample is warranted.

## 1. Introduction

Falling on the stairs, especially in descending, could result in severe injuries [1, 2] or even death [3]. Descending stairs demands a high level of neuromuscular control, especially of the lower limbs [1, 2]. It also involves cognition and attention to evaluate the configuration of the stairs and to integrate information from various sensory systems [1, 2, 4]. As cognition is involved in descending stairs, an additional cognitive task (dual-tasking) may interfere with the maneuver. A previous study has demonstrated altered foot clearance when stepping down among healthy older adults using a Stroop test [5]. Another study also found that, when compared with single-tasking, older adults made more errors in an auditory Stroop test when stepping down [6]. Indeed, it has been suggested that attentional distraction is one of the most common situations in which stair-related falls occur [7].

Negotiating stairs is an important indicator of independence among community-dwelling stroke survivors [8] and is considered as one of the most difficult activities in their daily life [9]. Impaired cognition and physical functioning after stroke may impair stairs negotiation ability. Stroke survivors descend stairs slower [10] and with greater lower limb strength demands [11] than their healthy counterparts. An additional cognitive task while stepping down, which may be encountered in daily life, may make the already demanding movement even more challenging. Previous auditory Stroop test results show that more attentional resources are needed in such a dual-tasking condition [6, 12]. Given the severity of the injuries caused by stair-related falls and the increased physical and cognitive demands when descending stairs, a cost-effective exercise, which will enhance the dual-tasking ability of stroke survivors when stepping down, is needed.

Tai Chi is a traditional Chinese martial art which, in recent decades, has been employed with different populations as an exercise to promote healthy aging and rehabilitation. Scientific studies have supported Tai Chi's training effects in terms of physical function and its utility in fall prevention among community-dwelling older adults [13–15] and stroke survivors [16–18]. Tai Chi movements demand proper posture, accurate joint positioning, constant weight shifting between the two legs, and eccentric control of the lower limbs. Significant improvements in lower limb strength [15] and neuromuscular control [19, 20] are observed after Tai Chi training, and both are important in safe and controlled stair descent. In addition, Tai Chi is considered a mind-body exercise. When practicing Tai Chi, attentions must be sustained to properly sequence the forms and to monitor the accuracy of the movements. The advantageous effects of Tai Chi training on cognitive functioning are demonstrated from prior studies [21, 22]. The involvement of cognition while performing the physical movement and maintaining balance gives Tai Chi a dual-tasking character. Indeed, a previous randomized controlled trial has illustrated improved dual-tasking performance among community-dwelling older adults after Tai Chi training. The subjects made fewer errors in an auditory Stroop test and swayed less when stepping down in a dual-tasking condition after the intervention [23].

Beyond the work of Lu and colleagues [23], however, there has been no similar study published addressing stroke survivors. This study was therefore designed to explore the effect of Tai Chi training on dual-tasking performance with such a population. Previous evidence with the training effects of Tai Chi on cognition, physical ability, and dual-tasking performance with healthy older adults suggests that dual-tasking performance that involves stepping down should be similarly improved among stroke survivors. We therefore hypothesized the following:

1. The cognitive and physical task performance under both single-tasking and dual-tasking conditions would be improved after Tai Chi training.
2. The performance in the Tai Chi group would be better than that of the conventional exercise group after the intervention period, owing to the dual-tasking features of Tai Chi and the lack of such characteristics in conventional exercises.

## 2. Methods

### 2.1. Participants

Subjects were recruited from patient self-help groups and hospitals in Hong Kong by distributing leaflet. So the subjects were basically self-selected, but the following inclusion criteria were applied: (1) aged 50 or above, (2) diagnosed with stroke six or more months previously, (3) able to perform a stepping down maneuver without any physical assistance, and (4) able to follow instructions in Cantonese. The exclusion criteria were the following: (1) any neurological disease other than stroke, (2) severe visual or hearing impairment, (3) a score of less than 18 on the Cantonese version of the Mini-Mental Status Examination (MMSE) [24], or (4) any major surgery or severe musculoskeletal injury during the previous six months. The study was approved by the Ethics Committee of the Hong Kong Polytechnic University. Written informed consent was obtained from each subject after the aims and procedures of the research had been explained. The assessment was carried out in the Department of Rehabilitation Sciences of the Hong Kong Polytechnic University, while the intervention was conducted at the university and in local community centers.

### 2.2. Intervention

The intervention was carried out between October 2014 and December 2016. After screening for eligibility, the subjects were stratified according to their gender and age (aged 50–59, 60–69, 70–79, or >80 years). The subjects were then randomized into Tai Chi, conventional exercise, or control groups within their stratified group.

#### 2.2.1. Tai Chi Group

Subjects in the Tai Chi group were taught a modified 12-form Yang-style Tai Chi (Table 1). The forms were selected according to the needs of stroke survivors by a senior physiotherapist and a Tai Chi master with more than 30 years of teaching experience. The training was instructed by another physiotherapist who is also an experienced Tai Chi practitioner. The Tai Chi training emphasized maintaining proper posture, eccentric control of the lower limbs, and increasing weight-bearing on the affected leg by continuous weight shifting. The subjects were encouraged to memorize the sequence of the forms and to monitor their own movement while practicing. There were end-of-training tests in which the participants were required to perform the 12 forms on their own so as to maximize the cognitive involvement and dual-tasking features of the exercise.

When learning a new form which challenged balance, such as Kick with Heels, physical support was provided initially to ensure subjects' safety. They initially performed those forms by leaning against the wall and holding the back of a chair, with the instructor standing by to provide assistance if necessary. The support was gradually diminished according to the subjects' standing ability. The class size was limited to 10 participants for close supervision. Rest periods were allowed whenever needed. Subjects in this group attended two one-hour training sessions each week, with a total of 24 sessions. There was a 10-minute warm-up at the beginning of each session and a 5-minute cooldown at the end. The subjects were encouraged to practice outside the class for 30 minutes at least once a week. Exercise log-books were given to the subjects for recording their self-practice.

#### 2.2.2. Conventional Exercise Group

Conventional exercises, which included upper and lower limbs mobilization, stretching, and muscle strengthening with elastic bands, as well as walking, were conducted in this group. Most of the exercises were performed in sitting except for some of the lower limb and walking exercises. Unlike the training in the Tai Chi group, all the exercises employed in this group are of single-tasking feature, which is focusing on the physical movements only. The subjects were not required to memorize the sequence of the exercises. Instead, they simply followed the movements of the instructor who was the same one who taught in the Tai Chi group. Frequency and time of the training of the conventional exercise group were the same as those of the Tai Chi group. Subjects were also given exercise log-books for recording the self-practice.

#### 2.2.3. Control Group

No training was provided to the control group during the 12-week intervention period. They continued their regular activities. After the completion of all the assessments, Tai Chi training was given to the subjects in this group.

### 2.3. Assessment

Demographic data, including age, gender, height, and weight, were collected at baseline. Information related to the stroke, such as the number, side, and type of stroke and the time since the onset, were also recorded. Physical functioning was assessed with the Timed Up-and-Go Test, the Berg Balance Scale (BBS), and functional reach. Cognitive ability was tested with the MMSE. All of the subjects were required to notify the research personnel of any change in their physical or medical condition, medication, exercise patterns, falls, and any injury during the study period.

A single cognitive task (an auditory Stroop test), a single physical task (stepping down), and dual-tasking were assessed before (preassessment), after (postassessment), and one month after (follow-up assessment) the intervention period. The order in which the three tasks were tested was randomized, but the sequence for any one subject was the same in all three assessments. The trained research assistant who conducted all the tests was blinded to the group assignments. Another researcher assisted for safety and operating the computer.

#### 2.3.1. Single Cognitive Task

An auditory Stroop test was used to quantify the executive function [25, 26]. Two Cantonese words, “high” and “low,” were pronounced with high and low pitches, resulting in four sound combinations. A switch with two buttons representing the high and low pitches was given to the subjects. They were instructed to ignore the meaning of the word but respond to the pitch of the sound by pressing the corresponding button with the thumb of the less-affected hand as quickly and as accurately as possible. The test was conducted while sitting. Each combination was repeated three times in random order, resulting in 12 trials.

A composite score was calculated as the outcome measure using the following formula [27]:

(1) $$\begin{array}{c} \text{composite score}=\frac{\text{accuracy}\left(\%\right)}{\text{averaged reaction time}\left(s\right)}. \end{array}$$

Accuracy was defined as the number of correct responses divided by the total number of trials. The reaction time was defined as the period between the appearance of the sound and pressing the button by the subject. Averaged reaction time was used to calculate the composite score. The parameters were recorded with a customized LabVIEW program (Version 8.6, National Instruments Corp., Austin, TX, USA).

#### 2.3.2. Single Physical Task

**Figure 1:**
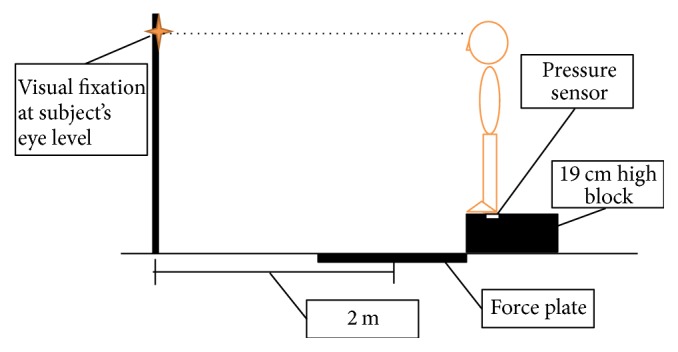
Setting of the stepping down task.

Stepping down was employed as the physical task. A force plate (model OR6-5-1000, Advanced Mechanical Technologies Inc., Newton, MA, USA) was positioned in front of a 19 cm high block, with a visual fixation placed two meters away from the center of the force plate at the subject's eye level to standardize the visual attention (Figure 1). Each subject was asked to stand in bare feet with the toes at the edge of the block and the legs apart at shoulder width. They then stepped down from the block, leading with the less affected leg. The movement ended when both feet were touching the force plate. The instructions called for the movement to be fast but as stably as possible. A research assistant stood next to the subject during the test to ensure safety. The task was repeated three times.

The force plate measured the sway of the center of pressure (CoP) during the stepping down movement. Data were captured as the leading leg reached the force plate, when the vertical ground reaction force exceeded zero, and the subsequent five seconds were used in the statistical analysis. The data were resampled from 1000 Hz to 125 Hz and filtered with a second-order Butterworth low-pass filter at a cut-off frequency of 10 Hz using a customized MATLAB program (Version 8.3, the MathWorks, Inc., Natick, MA, USA). The outcome measures were the anteroposterior (CoP-AP) and mediolateral (CoP-ML) sway amplitudes and the average sway velocity (VCoP), which was calculated by dividing the total CoP travel distance by five seconds. The data were normalized using each subject's height. Averages over three trials were employed in the data analysis.

#### 2.3.3. Dual-Tasking

Dual-tasking was assessed by combining the cognitive and the physical tasks. The setting was the same as that of the single stepping down test. A pressure sensor (model FSR406, Interlink Electronics, CA, USA) was placed under the calcaneus of the leading leg at the starting position. The auditory Stroop test was triggered when the leading leg was lifted and unloaded the pressure sensor. Each auditory Stroop test combination was examined once, resulting in four trials. The subjects were told that both the cognitive and physical tasks were of equal importance. The outcome measures used in the single-tasking conditions were applied in the dual-tasking.

The test-retest reliabilities were assumed to be the same as those previously measured elsewhere with 10 stroke survivors [12]. They were quantified in terms of intraclass correlation coefficients (ICCs). The reliability of the cognitive task in single- and dual-tasking was thus taken as ICC (3, 12) = 0.983 and ICC (3, 4) = 0.789, respectively. The ICC (3, 3) values of stepping down ranged from 0.681 to 0.941 in single-tasking, while the ICC (3, 4) values ranged from 0.744 to 0.913 in dual-tasking.

### 2.4. Statistical Analysis

The data analysis was conducted with the Statistical Package for the Social Sciences (SPSS, version 23, IBM Corp., Armonk, NY, USA). The baseline demographic data were compared among the three groups using one-way analysis of variance (ANOVA) for the continuous data and chi-squares tests for the categorical data. Two-way mixed ANOVAs (3 × 3) were conducted to determine the significance of any group effect (between-subject), time effect (within-subject), and any interaction effect (group × time) for each measure. When a significant group effect emerged, one-way ANOVA and follow-up post hoc analyses were employed to examine the significance of any between-subject differences at each assessment period. For a significant time effect, repeated-measures ANOVA with contrast analyses was conducted to investigate the within-subject changes in each intervention group. The level of significance was set at 0.05, with Bonferroni adjustments applied in all follow-up analyses. Missing data were treated by carrying the last observation forward in keeping with the intention-to-treat approach.

## 3. Results

**Figure 2:**
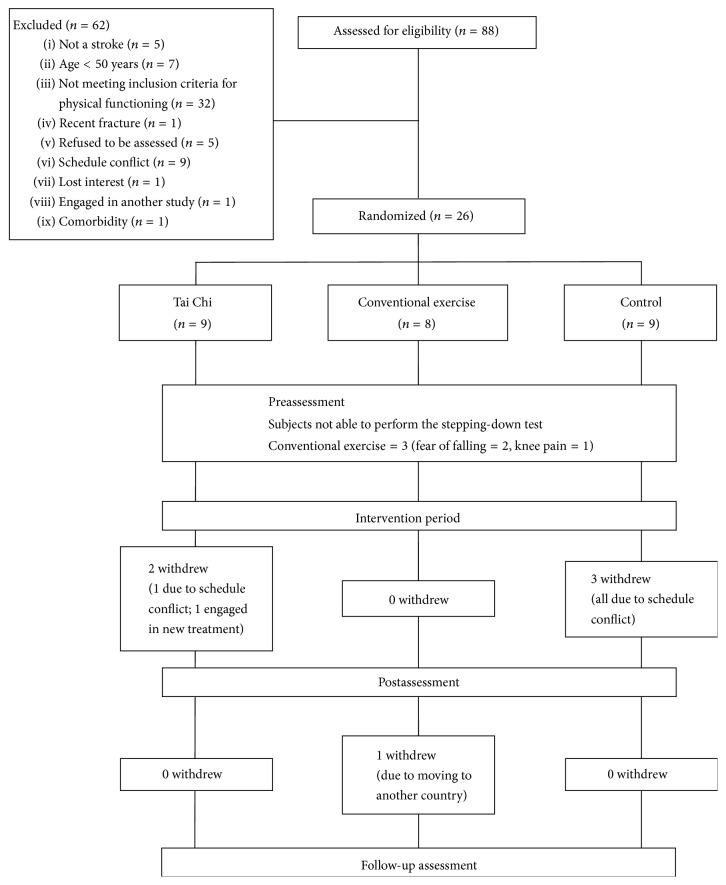
CONSORT diagram.

Eighty-eight subjects were enrolled in the study. After screening for eligibility, a total of 26 subjects were randomized into the Tai Chi (*n* = 9), conventional exercise (*n* = 8), or control (*n* = 9) group. Three of the subjects in the conventional exercise group proved unable to perform the stepping down test due to fear of falling (*n* = 2) or knee pain (*n* = 1) during the assessment, so there were only five subjects in that group. Another five subjects eventually withdrew (Tai Chi: *n* = 2, controls: *n* = 3) due to schedule conflicts (*n* = 4) or having started a new treatment (*n* = 1). Another subject from the conventional exercise group dropped out after the postassessment because of moving to another country.  Figure 2 shows a CONSORT diagram for the study.

### 3.1. Baseline Comparison and Adherence

Table 2 presents demographic data describing the subjects. There was no significant difference in baseline characteristics among the three groups. The attendance of the Tai Chi group and that of the conventional exercise group were 94.0% and 78.3%, respectively. No adverse effect related to the intervention or the assessment was reported.

### 3.2. Single-Tasking Condition

The two-way mixed ANOVA showed no significant interaction effect [*F*(4,40) = 0.183; *p* = 0.946], group effect [*F*(2,20) = 0.491; *p* = 0.619], or time effect [*F*(2,40) = 2.985; *p* = 0.062] in the composite scores on the auditory Stroop test. Likewise, no significant effects were found in any of the stepping down test results in the single-tasking condition (*p* > 0.05) (Table 3).

### 3.3. Dual-Tasking Condition

Table 4 displays the results of the two-way mixed ANOVA of the cognitive and physical task performance in the dual-tasking condition.

There was a significant interaction between group and time in the composite scores of the auditory Stroop test [*F*(4,40) = 4.136; *p* = 0.007]. Improvement was observed in the Tai Chi group from the preassessment (64.6 ± 22.7) to the postassessment (91.9 ± 19.2), but the change was not significant at the study's 5% level of confidence (*p* = 0.054). The score continued to increase, however, reaching 94.4 ± 20.6 in the follow-up assessment, a significant improvement compared with the preassessment (*p* = 0.036). The composite score of the Tai Chi group was also significantly higher than that of the conventional exercise group in the follow-up assessment (*p* = 0.013). None of the other within-subject and between-subject comparisons displayed statistically significant differences (*p* > 0.05).

Apart from the cognitive task, the CoP-ML also demonstrated a significant interaction effect [*F*(4,40) = 3.162; *p* = 0.024]. However, the group effect was not significant in all testing periods (*p* > 0.05). In the conventional exercise group, CoP-ML tended to decrease from the preassessment (93.6 ± 29.1) through to the follow-up assessment (51.8 ± 16.7), but a follow-up analysis showed that the changes were not statistically significant (*p* = 0.051). No significant time effect was found in the Tai Chi group and the control group (*p* > 0.05).

The other two observations from the stepping-down task, the CoP-AP and VCoP, showed no significant interaction effect [CoP-AP: *F*(4,40) = 1.315 and *p* = 0.287; VCoP: *F*(4,40) = 1.308 and *p* = 0.292], group effect [CoP-AP: *F*(2,20) = 0.113 and *p* = 0.894; VCoP: *F*(2,20) = 0.194 and *p* = 0.825], or time effect [CoP-AP: *F*(2,40) = 1.429 and *p* = 0.252; VCoP: *F*(2,40) = 1.596 and *p* = 0.222].

## 4. Discussion

This pilot study investigated the effect of Tai Chi training on dual-tasking performance that involved stepping down. The expectation that Tai Chi training would improve both single-tasking and dual-tasking performance was partially supported. A significant enhancement in the average composite score of the auditory Stroop test was observed from the preassessment to the follow-up assessment without compromising physical task performance. However, no significant change in single-tasking performance was observed in the Tai Chi group or among the others. But the Tai Chi group's significantly higher average composite score at the follow-up assessment compared with the conventional exercise group supports the idea that Tai Chi training on dual-tasking is better than the conventional exercise.

Our previous studies on dual-tasking which involved an auditory Stroop test and stepping down have shown motor-related cognitive interference in dual-tasking among healthy older adults [6] and stroke survivors [12]. In this study, the stroke survivors showed a significant increase in their average composite score on the cognitive task in dual-tasking after 12 weeks of Tai Chi training (from 64.6 ± 22.7 in the preassessment to 94.4 ± 20.6 in the follow-up assessment). The average composite score in dual-tasking (Table 4) during follow-up assessment was comparable to that in single-tasking (Table 3) and even to that of the healthy older subjects under dual-tasking condition that involves stepping down [12].

As there has previously been no study published pertaining to the training effect of Tai Chi on dual-tasking performance among stroke survivors, the results of this study were compared with those of the prior studies with healthy older adults [23, 28, 29]. Wayne and his colleagues investigated the effect of Tai Chi training on the dual-tasking performance of healthy older adults (*n* = 31, mean age = 63.9 ± 8.0 years) using a serial subtraction task and walking ability. The results illustrated significantly higher gait speed under dual-tasking conditions after six months of Tai Chi training [29]. Lu and colleagues also found a significant improvement in dual-tasking performance after 16 weeks of Tai Chi training with healthy older adults. There was a significant reduction in error rate in an auditory Stroop test in the dual-tasking condition. Their subjects also swayed significantly less when stepping down with a concurrent auditory Stroop test [23]. In contrast, no obvious change was revealed in an earlier study [28]. In that study, two different dual-tasking conditions were employed: (1) auditory stimulation with a sensory organization test and (2) visual stimulation with avoiding obstacles while walking. Older adults (*n* = 8, mean age = 72.2 ± 7.7 years) were trained in Tai Chi twice a week, 1.5 hours per class, for 12 weeks. The different results from those previous studies could be due to the instruction method during the Tai Chi training. In the study by Hall and colleagues [28], rich visual cues were provided so that the participants only needed to follow the instructor's movements during practice. That presumably reduced active cognitive processing among the subjects, thus decreasing the dual-tasking impact of Tai Chi. In this study, the subjects were explicitly instructed to concentrate and memorize the forms and their sequencing. They were also enjoined to monitor and adjust their movement during practice. In addition, end-of-training tests were anticipated. These techniques may have increased the participants' involvement and the cognitive functioning, which should enhance dual-tasking performance.

In dual-tasking, deterioration of the performance of either task or both tasks can be explained by competition for attentional resources, which is considered a result of (1) insufficient attentional resources available to perform both tasks simultaneously, (2) inability to properly allocate or switch attentional resources between the two tasks, (3) increased attentional resources to carry out one individual task, or (4) a combination of these factors [30]. Theoretically, dual-tasking performance can be improved by modifying these factors. One explanation for the enhanced dual-tasking performance after Tai Chi training could be the increased attentional resources available to perform both the cognitive and physical tasks simultaneously. A previous cross-sectional study using fMRI showed that Tai Chi practitioners (*n* = 22, mean age = 52 ± 6 years, and mean experience = 14 ± 8 years) had greater cortical thickness at the middle frontal sulcus when compared with nonpractitioners (*n* = 18; mean age = 54 ± 6 years) [31]. That area is responsible for executive function, attention, working memory, and processing spatial information [32–34]. The cortical thickness of the right precentral gyrus, which is activated during spatial processing, space and motion perception and imagery, orientation of attention, motor-skill learning [34], and the execution of motor task [35], is also found to be greater in Tai Chi practitioners [31], as is that of the left medial occipitotemporal sulcus and the lingual sulcus, which are responsible for retrieving and integrating spatial information [34, 36, 37]. As Tai Chi practice involves continuous planning of movement as well as monitoring of posture and the relative position of the body and the external environment, long-term practice may alter the structure of the corresponding brain regions. Studies seeking causal relationship between the effects of Tai Chi practice and changes in brain structure and attentional resources are warranted.

Another possible explanation for the enhanced dual-tasking performance after Tai Chi training found in this study might be an improved ability to allocate or shift attentional resources between the two tasks. Fong and his colleagues have compared task-switching performance among older Tai Chi practitioners (*n* = 16, mean age = 67.3 ± 4.9 years, and average experience = 13.6 ± 8.6 years), older adults with sedentary lifestyle (*n* = 16; mean age = 68.9 ± 4.3 years), and young adults (*n* = 16; mean age = 22.4 ± 2.6 years) using event-related potentials [38]. The results showed higher P300 amplitudes among those with Tai Chi experience compared with the older sedentary adults, which is positively related to the ability to allocate attentional resources to different tasks [39]. The amplitude was even comparable to that of the younger subjects. When practicing Tai Chi, the practitioner should maintain attention and shift the focus between cognitive processing and physical movement in order to memorize and plan the forms while maintaining balance. The proportion of attentional resources allocated to the cognitive and physical components may differ with the complexity of the forms and one's ability to perform them. For example, in Golden Rooster Stands on One Leg, a form which involves single-leg standing but only simple upper limb movement, more attentional resources should be allocated to the physical task of maintaining balance. By contrast, the form Wave Hands in Clouds requires coordinated movement of arms but a rather stable posture, so attentional resources should be allocated preferentially to cognitive processing. For those forms with complex motions while also challenging balance, such as Brush Knee and Twist Step, or Step Back and Whirl Arms, attentional resources must be shared more equally between the cognitive and physical functions. In this connection, there has been no published study investigating any causal relationships between Tai Chi training and neuroplasticity or neurogenesis in either healthy older adults or chronic stroke survivors. The topics of brain activity and the allocation of attentional resources during Tai Chi practice are also understudied.

In this study, no significant changes in the performance of the single cognitive or single physical task were observed after Tai Chi training. This may imply that the improved dual-tasking performance is probably not attributable to a decreased demand for attentional resources in the individual tasks. Indeed, absence of any significant change in the single-tasking conditions was not expected, as it has been shown that Tai Chi training benefits both cognitive and physical abilities. Prior studies have revealed significantly better executive functioning, such as attention [21] and working memory [22], in healthy older adults after Tai Chi training. For the physical ability, a biomechanical study demonstrated that during Tai Chi practice there is a high level of prolonged eccentric control and cocontraction of the lower limb muscles [40], which are important factors in lowering the body and stabilizing the lower limb joints when stepping down [41]. A previous randomized controlled trial also exhibited significantly stronger eccentric knee extensor strength after Tai Chi training [15].

Notwithstanding the lack of statistically significant changes in the single-tasking conditions, the average composite score in the auditory Stroop test improved from 85.9 ± 28.6 in the preassessment to 96.2 ± 26.7 in the follow-up assessment in the Tai Chi group. The normalized sway amplitude of the CoP in the mediolateral direction also decreased from 73.9 ± 41.6 in the preassessment to 52.7 ± 17.0 after the Tai Chi training in single-tasking (Table 3). The trend suggests that a longer training period might allow for greater improvement. Further studies should be conducted to evaluate the effects of long-term Tai Chi training on executive function and stepping down performance among stroke survivors. On the other hand, a recent study suggests that complexity-based measure of postural sway is a sensitive parameter to reflect the effect of Tai Chi training on postural control. The sway was found to be higher among experienced Tai Chi practitioners and healthy older adults after six months of Tai Chi training, which may be attributed to the improved dynamic and adaptive postural control [42]. Further research employing this parameter to reflect the change after Tai Chi training is worth conducting.

The expectation that the training effect of Tai Chi would be better than that of the conventional exercises was suggested. The composite score in the auditory Stroop test under dual-tasking was significantly higher in the Tai Chi group than in the conventional exercise group in the follow-up assessment. The subjects in the conventional exercise group were only asked to follow the instructor's movement instead of memorizing the sequence of exercise. The lack of cognitive involvement during the conventional exercise training may have contributed to the insignificant change in dual-tasking performance after the intervention. Indeed, current evidence is still insufficient to support the effect of physical exercise alone on improving dual-tasking performance [43]. That group's lower composite score in the auditory Stroop test and less CoP sway when stepping down under dual-tasking suggest that more attentional resources were being allocated to the physical task rather than the cognitive task during the follow-up assessment compared with the initial one. That should improve safety in negotiating stairs while dual-tasking, but it may also imply that the attentional resources were insufficient for performing the tasks concurrently [30]. On the other hand, the Tai Chi subjects showed significant improvement in dual-tasking performance. The effect of Tai Chi training on dual-tasking performance is therefore postulated to be better than that of the conventional exercises tested. However, the strategy adopted by the subjects after the conventional exercise training was still appropriate for maintaining stability.

It is important to remind readers that the sample size of this study was small and the dropout rate was relatively high. Results of this study are only considered as preliminary and future studies with a larger number of subjects should be conducted. Post hoc sample size calculations show that a total of 45 subjects would be needed to demonstrate a significant interaction effect in the physical task (*α* = 0.05, power = 0.9, and effect size = 0.25). Although the number of subjects who participated in this study was lower than that of the calculated sample size, significant improvement in dual-tasking performance was still found. This may support the value of Tai Chi training on dual-tasking performance in stroke survivors. Another limitation was the relatively high level of cognition and physical functioning of the subjects (mean MMSE = 28.0 ± 2.1; BBS = 52.1 ± 2.9). As the dual-tasking that involves stepping down is challenging to stroke survivors, those who have a lower cognitive and physical abilities were excluded from this study, which may result in selection bias and limit the generalizability of the study's results. Further studies on stroke survivors with a lower level of cognitive and physical functions are warranted.

## 5. Conclusions

This is the first study investigating the effect of Tai Chi training on dual-tasking performance which involved stepping down among stroke survivors. These results suggest that 12 weeks of Tai Chi training (twice a week for an hour) is feasible and safe for stroke survivors and that it can be incorporated into rehabilitation programs to improve dual-tasking performance. Also, the significant results found during the follow-up period imply that continued Tai Chi practice should be encouraged after completion of the 12-week training period. In addition, subjects should be encouraged to learn the Tai Chi patterns rather than just following the instructor's movements. The results also show that Tai Chi training can better improve the cognition in dual-tasking condition compared to conventional exercises. However, further studies with a larger sample and involving less able subjects are needed. Moreover, the mechanisms underlying the effects demonstrated here remain unclear and are worthy of further investigation.

## Figures and Tables

**Table 1:** Name and training specificity of the 12 Yang-style Tai Chi forms tested.

	Name	Proper posture	Eccentric control	Turning	Forward stepping	Backward stepping	Sideways stepping	Single-leg standing	Relaxation	Concentration
(1)	Commencing	v							v	v

(2)	White Crane Spreads its Wings	v	v							v

(3)	Brush Knee and Twist Step	v		v	v					v

(4)	Hands Strum the Lute	v	v			v				v

(5)	Step Back and Whirl Arms	v				v				v

(6)	Work at Shuttles on Both Sides	v		v	v					v

(7)	Needle at the Sea Bottom	v	v	v	v					v

(8)	Wave Hands in Clouds	v		v			v			v

(9)	Golden Rooster Stands on One Leg	v						v		v

(10)	Kick with Heel	v						v		v

(11)	Strike Opponent's Ears with Both Fists	v							v	v

(12)	Closing	v							v	v

**Table 2:** Demographic and clinical characteristics of the subjects.

	Tai Chi (*n* = 9)	Conventional exercise (*n* = 5)	Control (*n* = 9)	*p* value
Age (year)	63.9 ± 6.1	63.2 ± 9.7	63.2 ± 6.0	0.975
Gender (male : female)	5 : 4	3 : 2	4 : 5	0.827
Height (cm)	161.4 ± 5.2	160.4 ± 5.4	162.6 ± 8.2	0.836
Weight (kg)	59.6 ± 4.5	57.4 ± 12.3	61.7 ± 16.8	0.821
Education (year)	8.4 ± 2.1	9.7 ± 3.2	10.3 ± 4.7	0.624
MMSE score	29.2 ± 0.4	27.0 ± 1.9	27.3 ± 2.7	0.074
BBS score	51.0 ± 2.9	50.3 ± 3.2	54.2 ± 1.7	0.074
Functional reach (cm)	23.9 ± 3.7	23.9 ± 8.3	32.1 ± 9.0	0.133
TUGT (sec)	14.5 ± 4.6	12.2 ± 2.1	12.3 ± 5.1	0.661
Outdoor walking aid (unaided : stick)	3 : 6	3 : 2	4 : 5	0.626
Chronicity (year)	3.3 ± 3.0	8.8 ± 7.9	4.6 ± 3.0	0.103
Number of strokes				
1	7	5	8	0.485
2	2	0	1	
Type of stroke (ischemic : hemorrhage : mixed)	7 : 1 : 1	2 : 3 : 0	8 : 1 : 0	0.140
Side of stroke (right : left)	5 : 4	3 : 2	8 : 7	0.254
Attendance (%)	94.0 ± 7.6	78.3 ± 20.7	N/A	0.091
Self-practice during the intervention period (min/week)	67.5 ± 44.2	145.1 ± 114.7	N/A	0.090
Self-practice during the follow-up period (min/week)	58.0 ± 52.2	10.0 ± 17.3	N/A	0.184

Values are in mean ± SD. MMSE: Mini-Mental Status Examination (Cantonese version); BBS: Berg Balance Scale; TUGT: Timed Up-and-Go Test.

**Table 3:** Results of two-way mixed ANOVA on single-tasking condition.

	Tai Chi			Conventional exercise			Control			*F*-value (*p* value)		
	Pre	Post	FU	Pre	Post	FU	Pre	Post	FU	Between-subject	Within-subject	Interaction
CS	85.9 ± 28.6	93.4 ± 21.7	96.2 ± 26.7	99.5 ± 17.3	108.0 ± 30.6	106.5 ± 19.5	93.8 ± 23.6	99.5 ± 19.4	98.2 ± 31.1	0.491 (0.619)	2.985 (0.062)	0.183 (0.946)
CoP-AP	80.4 ± 36.8	79.5 ± 17.4	85.6 ± 14.4	76.0 ± 18.6	99.6 ± 28.0	81.9 ± 21.5	83.7 ± 25.8	81.4 ± 24.9	77.4 ± 27.5	0.100 (0.905)	0.863 (0.411)	1.336 (0.279)
CoP-ML	73.9 ± 41.6	52.7 ± 17.0	68.8 ± 27.1	84.4 ± 15.7	79.3 ± 15.5	56.7 ± 21.9	71.9 ± 31.3	59.7 ± 34.7	77.2 ± 38.0	0.221 (0.803)	1.644 (0.206)	1.5289 (0.212)
VCoP	65.8 ± 36.1	62.0 ± 19.6	62.3 ± 11.5	71.9 ± 14.1	76.2 ± 27.1	65.9 ± 16.1	68.1 ± 22.2	59.9 ± 22.6	66.0 ± 23.8	0.264 (0.771)	0.462 (0.608)	0.654 (0.608)

Values are in mean ± SD or *F-*value (*p* value). Pre: preassessment; Post: postassessment; FU: follow-up assessment; CS: composite score on the auditory Stroop test; CoP-AP: normalized CoP sway in the anteroposterior direction; CoP-ML: normalized CoP sway in the mediolateral direction; VCoP: average CoP sway velocity.

**Table 4:** Results of two-way mixed ANOVA on dual-tasking condition.

	Tai Chi			Conventional exercise			Control			*F*-value (*p* value)		
	Pre	Post	FU	Pre	Post	FU	Pre	Post	FU	Between-subject	Within-subject	Interaction
CS	64.6 ± 22.7	91.9 ± 19.2	94.4 ± 20.6	66.9 ± 26.4	64.4 ± 16.0	55.7 ± 11.1	86.1 ± 38.2	82.6 ± 20.7	72.9 ± 26.1	2.069 (0.153)	0.920 (.407)	4.136 (0.007)^a,b,c^
CoP-AP	89.6 ± 46.7	86.0 ± 25.3	91.2 ± 18.6	97.1 ± 30.5	97.0 ± 35.5	72.1 ± 24.2	88.8 ± 25.5	80.1 ± 32.3	80.2 ± 31.9	0.113 (0.894)	1.429 (0.252)	1.315 (0.287)
CoP-ML	72.7 ± 40.0	59.9 ± 18.9	71.4 ± 18.8	93.6 ± 29.1	80.7 ± 14.6	51.8 ± 16.7	65.5 ± 26.9	56.1 ± 30.0	77.4 ± 36.4	0.290 (0.751)	1.860 (0.169)	3.162 (0.024)^a^
VCoP	66.2 ± 39.8	64.5 ± 16.7	64.8 ± 10.5	82.8 ± 26.1	71.6 ± 24.2	59.8 ± 12.5	66.4 ± 20.0	61.5 ± 21.0	67.7 ± 23.0	0.194 (0.825)	1.596 (0.222)	1.308 (0.292)

Values are in mean ± SD or *F-*value (*p* value). Pre: preassessment; Post: postassessment; FU: follow-up assessment; CS: composite score on the auditory Stroop test; CoP-AP: normalized CoP sway in the anteroposterior direction; CoP-ML: normalized CoP sway in the mediolateral direction; VCoP: average CoP sway velocity. ^a^Statistical significant interaction (*p* < 0.05); ^b^statistical significant difference between the Tai Chi and conventional exercise groups in the follow-up assessment (*p* < 0.05); ^c^statistical significant change from the preassessment to the follow-up assessment in the Tai Chi group (*p* = 0.036).
